# Antibacterial Activity of a Bioactive Tooth-Coating Material Containing Surface Pre-Reacted Glass in a Complex Multispecies Subgingival Biofilm

**DOI:** 10.3390/pharmaceutics15061727

**Published:** 2023-06-14

**Authors:** Caio Junji Tanaka, José Augusto Rodrigues, João Marcos Spessoto Pingueiro, Tatiane Tiemi Macedo, Magda Feres, Jamil Awad Shibli, Bruno Bueno-Silva

**Affiliations:** 1 School of Dentistry, Mogi das Cruzes University, Mogi das Cruzes 08780-911, Sao Paulo, Brazil; caiotanaka@umc.br; 2 Dental Research Division, Department of Periodontology and Restorative Dentistry, Guarulhos University UNIVERITAS/UNG, Guarulhos 07011-010, Sao Paulo, Brazil; spessoto.jm@gmail.com (J.M.S.P.); tatianet92@gmail.com (T.T.M.); mferes@ung.br (M.F.); jshibli@ung.br (J.A.S.); brunobue@gmail.com (B.B.-S.); 3 School of Dentistry, Sao Judas Tadeu University, Sao Paulo 05503-001, Sao Paulo, Brazil; 4 Department of Oral Medicine, Infection, and Immunity, Division of Periodontology, Harvard School of Dental Medicine, Boston, MA 02115, USA; 5 Department of Biosciences, Piracicaba Dental School, University of Campinas—UNICAMP, Piracicaba 13414-903, Sao Paulo, Brazil

**Keywords:** PRG filler, resin coating, varnish, bioactivity, biofilms, antimicrobial activity, caries prevention, periodontal disease, inflammation, ion release

## Abstract

Bioactive materials were developed with the ability to release fluoride and provide some antimicrobial potential, to be widely used in dentistry today. However, few scientific studies have evaluated the antimicrobial activity of bioactive surface pre-reacted glass (S-PRG) coatings (PRG Barrier Coat, Shofu, Kyoto, Japan) on periodontopathogenic biofilms. This study evaluated the antibacterial activity of S-PRG fillers on the microbial profile of multispecies subgingival biofilms. A Calgary Biofilm Device (CBD) was used to grow a 33-species biofilm related to periodontitis for 7 days. The S-PRG coating was applied on CBD pins from the test group and photo-activated (PRG Barrier Coat, Shofu), while the control group received no coating. Seven days after treatment, the total bacterial counts, metabolic activity, and microbial profile of the biofilms were observed using a colorimetric assay and DNA–DNA hybridization. Statistical analyses were applied; namely, the Mann–Whitney, Kruskal–Wallis, and Dunn’s post hoc tests. The bacterial activity of the test group was reduced by 25.7% compared with that of the control group. A statistically significant reduction was observed for the counts of 15 species: *A. naeslundii*, *A. odontolyticus*, *V. parvula*, *C. ochracea*, *C. sputigena*, *E. corrodens*, *C. gracilis*, *F. nucleatum polymorphum*, *F. nucleatum vincentii*, *F. periodonticum*, *P. intermedia*, *P. gingivalis*, *G. morbillorum*, *S. anginosus*, and *S. noxia* (*p* ≤ 0.05). The bioactive coating containing S-PRG modified the composition of the subgingival biofilm in vitro, thereby decreasing colonization by pathogens.

## 1. Introduction

Periodontitis is a chronic inflammatory disease associated with the interaction of different factors, resulting in a dysbiotic host–biofilm interaction that leads to the continued destruction of the tooth-supporting tissues, with progressive loss of insertion and apical migration of the junctional epithelium [1]. The pathological association of different species of bacteria, mostly of the orange and red complexes in subgingival biofilms, acts as the etiological factor for the onset of the disease [2,3]. The association of pathogenic bacteria results in the progressive destruction of the periodontium through the release of destructive factors and enzymes, which damage host cell membranes and extracellular matrixes to obtain nutrients for their growth [1,2]. Periodontal treatment consists of controlling infection; reducing the depth of the probing pocket; and increasing the clinical attachment level through plaque control, scaling, and root planing in order to mechanically remove the local pathogenic bacteria of the biofilm, allowing for a different recolonization and development of a new biofilm, preferably composed of bacteria associated with health, such as the bacteria of the green complex [2,3,4,5,6]. Adjunctive therapies, such as antiseptics, systemic antibiotics, and probiotics, are generally used to potentiate the effect of the mechanical treatment [1,6].

Antimicrobial activity is a desirable property for bioactive restorative materials and this property might also be used as new approach against periodontal bacteria [7], in an attempt to prevent the colonization of bacteria on the surface of restorations. Modifications of restorative materials to improve antimicrobial activity and provide bioactivity are proposed in the literature through the addition of different monomers, active substances, and ions [8,9,10].

Bioactive materials were developed with the ability to release fluoride and provide some antimicrobial potential, including glass ionomers, hybrid materials such as compomers, and adhesives and composite resins [11,12,13]. These materials may act by releasing fluoride around the margins of the restoration. The potential to release fluoride ions may inhibit the demineralization of the tissue and improve its remineralization, acting directly on the balance of the carious process [14]. Recently, a new class of bioactive materials has emerged, namely the giomers. Generally, they incorporate the restorative properties of resin composites, such as mechanical resistance and aesthetics, while ions are released and recharged as the fluoride in the glass ionomer cement improves defense against caries disease. Clinical studies suggest that mechanical strength, marginal adaptation, resistance to degradation, and postoperative sensitivity are similar to those of conventional composite resins and effectively reduce the frequency of secondary caries [10,15,16,17,18]. Giomer materials incorporate a surface pre-reacted glass (S-PRG) filler technology which represents an important advance in the field of ion-releasing dental materials merging bioactivity, biomimetics, and biocompatibility. Giomers are available as composite resins, sealants, and a protective coating sealant.

PRG Barrier Coat (Shofu Inc., Kyoto, Japan) is a protective material that coats the tooth surface with a resin sealant with a high amount of S-PRG filler. It can be used as a preventive strategy for patients with high caries risk. This material is indicated for preventing dental caries in children and in high-risk patients, such as orthodontic patients, the elderly, and special needs or handicapped patients, as well as for reducing dentin hypersensitivity by coating all dental surfaces. Furthermore, long-lasting results can be expected due to the continuous release and recharge of fluoride and other ions [19,20].

The S-PRG filler releases six ions, namely, fluoride (F), aluminum (Al), boron (B), silica (Si), strontium (Sr), and sodium (Na). These ions are able to act on the demineralization–remineralization balance and also on bacterial activity [18,21,22,23,24]. In vitro studies have shown the antimicrobial activity of an eluate of S-PRG filler by inhibiting the growth of *S. mutans*, *S. gordonii*, *P. gingivalis*, *Fusobacterium nucleatum*, and *C. albicans* [13,18,20,25,26,27,28,29,30]. These findings indicate that S-PRG filler has antimicrobial activity and could be indicated as a preventive strategy against caries disease and periodontal disease. S-PRG in eluate form is able to reduce the disruption of collagen in the periodontal tissue and reduce the infiltration of inflammatory cells, which produce oxidative stress, stunting, and inhibit virulence factors [31]. Thus, the use of S-PRG fillers may be beneficial in the reduction of pathogenic bacteria and contribute to oral health. Despite these promising and relevant results on caries and periodontal pathogens, no studies were found that test S-PRG filler’s bioactivity against a complex periodontal biofilm.

Therefore, this in vitro study evaluated the impact of a bioactive tooth-coating material with surface pre-reacted glass (S-PRG) on a multispecies subgingival biofilm with 31 periodontopathogenic species. The null hypothesis was that the active biomaterial would not impact the metabolic activity of the pathogenic biofilm (h0). 

## 2. Materials and Methods

### 2.1. Bioactive Tooth-Coating Material

The test product, PRG Barrier Coat (Shofu, Kyoto, Japan), was presented as two mixing vials: an activator (phosphonic acid monomers, methacrylic acid monomers, bis-MPEPP (2,20-bis [4-methacryloxy polyethoxyphenyl propane]), carboxylic acid monomers, TEGDMA (triethylene glycoldimethacrylate), a polymerization initiator and others), and a base composed of a mix of PRG fillers (aluminofluoro-borosilicate glass, 50–60%), methacrylic acid monomers, water, and others.

To prepare the material, one drop of the activator was added to the base and mixed. The mixture was painted on the surface of 3 pins in a single direction. The PRG barrier coat was left for 3 s and then the samples were photo-activated for 10 s with a LED curing unit. After light-curing, the uncured layer was gently removed with water and a moistened cotton ball.

### 2.2. Biofilm Formation

For the biofilm development [32], pure cultures of each bacterium were grown. Most species, including *Actinomyces* subsp., *Streptococcus* subsp., and *Fusobacterium* subsp., were grown under anaerobic conditions (85% N_2_, 10% CO_2_, and 5% H_2_), on tryptone soy agar (TSA) with sheep blood (5%). *Porphyromonas gingivalis* was grown on TSA with yeast extract enriched with hemin (1%), menadione (5%), and sheep blood (5%). The *Tannerella forsythia* was grown on TSA with yeast extract enriched with hemin (1%), menadione (5%), sheep blood (5%), and N-acetylmuramic acid (1%). Eubacterium was grown on fastidious anaerobic agar (FAA), also with sheep blood (5%). The bacteria used to form the subgingival biofilm are listed in Table 1.

After 48 h of growth on agar plates, all species were reallocated to conical tubes with a brain–heart infusion medium (BHI, Becton Dickinson, Sparks, MD, USA) supplemented with hemin (1%). After 24 h of growth in the BHI broth, the optical density (OD) at 600 nm was restricted to 0.1, which corresponded to 10^8^ cells/mL of each species. The individual cell suspensions were diluted and mixed to obtain a final biofilm inoculum containing 10^4^ cells/mL of each species.

The model of the multispecies biofilm was performed using a Calgary Biofilm Device (CBD). In each well of a 96-well plate (Nunc; Thermo Scientific, Roskilde, Denmark), 150 µL of the inoculum was added and a lid containing the polystyrene pins was used.

Immediately before incubating the Calgary devices in the medium containing the bacterial inoculum, three pins were covered with S-PRG and another three pins were left without any coating, which were used as negative controls. Since this material is new to the market, we could not find any other products that could be considered as positive controls.

The CBD was incubated under anaerobic conditions at 37 °C. Three days after incubation, the plate covers were transferred to new 96-well plates with fresh BHI broth with hemin (1%) and sheep’s blood (5%) for another four days. After seven days of biofilm development, the pins were removed. Metabolic activity and checkerboard DNA–DNA analyses were performed. Three independent experiments were carried out with five pins for each analysis.

### 2.3. Metabolic Activity of the Biofilm

The percentage of reduction in the biofilm’s metabolic activity was determined using 2,3,5-triphenyltetrazolium chloride (TTC) (catalog No. 17779, Analytical Fluka) and spectrophotometry. TTC was chosen to observe metabolically active and inactive cells. The white substrate was enzymatically reduced to 1,3,5-triphenyl red formazan (TPHP) by live bacterial cells through the activity of various dehydrogenases. The change in the color of the substrate was measured using spectrophotometry to observe the rate of reduction, used as an indirect measure of the bacteria’s metabolic activity. To quantify the metabolic activity of the biofilms, the pins were washed twice with a washing solution and transferred to plates with 200 µL per well of fresh BHI medium containing hemin (1%) with 10% TTC solution. The plates were incubated in anaerobic conditions at 37 °C for 24 h. The conversion of TTC was read at 485 nm using a fluorescence spectrophotometer.

### 2.4. Checkerboard DNA–DNA Hybridization

The suspensions of the biofilm contained in the plastic tubes were boiled in a water bath for 10 min and then neutralized by the addition of 0.8 mL of 5 M ammonium acetate. Each suspension of dental biofilm containing free DNA was deposited in one of the channels of a Minislot with 30 channels (Immunetics, Cambridge, MA, USA) and transferred to a positively charged nylon membrane (15 × 15 cm^2^) (Merstham Biosciences UK Limited, Buckinghamshire, UK). The last two of the Minislot channels were occupied by controls containing a mixture of the species of microorganisms investigated with probes, in concentrations corresponding to 10^5^ and 10^6^ cells. The membrane was removed from the Minislot 30 and the concentrated DNA was fixed by heating it for 20 min in an oven at 120 °C. The membrane was prehybridized for 1 h at 42 °C in a solution containing 50% formamide, 1% casein, 5× citrated saline solution (SSC) (1× SSC = 150 mM NaCl, Vetec Quimica Fina Ltda., Rio de Janeiro, RJ, Brazil), 15 M sodium citrate (JTBaker, Edo. De Méx., Mexico) at pH 7.0, 25 mM sodium phosphate (Na_2_HPO_4_, Labsynth, Diadema, Brazil) at pH 6.5, and 0.5 mg/mL of yeast RNA (Sigma, St. Louis, MO, USA), then positioned on a Miniblotter 45 (Immunetics, Cambridge, MA, USA) with the lines containing the DNA of the samples and the controls positioned perpendicularly to the channels of the apparatus. A DNA probe was added to each channel of the Miniblotter 45, diluted to approximately 20 ηg/mL in 130 mL of hybridization solution composed of 45% formamide, 5× SSC, 20 mM Na_2_HPO_4_ (pH 6.5), 0.2 mg/mL of yeast RNA, 10% dextran sulfate (Amersham, Stafford, UK), and 1% casein. Hybridization occurred within a minimum period of 20 h at 42 °C.

### 2.5. Detection of Species

After the hybridization period, the membrane was removed from the Miniblotter 45 (Immunetics, Boston, MA, USA) and washed for 40 min at 65 °C in an astringent solution composed of 1% SDS, 1 mM EDTA, and 20 mM Na_2_HPO_4_ to remove any probes that had not completely hybridized. Then the membrane was immersed for 1 h in a solution containing 1% maleic acid (C_4_H_4_O_4_, Vetec, Speyer am Rhein, Germany), 3 M NaCl, 0.2 M NaOH (Labsynth), 0.3% Tween 20 (Vetec), and 0.5% casein (pH 8.0), and then immersed for 30 min in the same solution containing the anti-digoxigenin antibody conjugated to alkaline phosphatase (Roche, Basel, Switzerland) at a concentration of 1:10,000. The membrane was then washed twice for 20 min in a solution of 0.1 M maleic acid, 3 M NaCl, 0.2 M NaOH, and 0.3% Tween 20 (pH 8.0), and washed once for 5 min in a solution of 0.1 M Tris HCl and 0.1 M NaCl (pH 9.5).

For the detection of signals, the membrane was incubated for 45 min at 37 °C in a detector solution containing a substrate of alkaline phosphatase and CDP-Star™ Detection Reagent (Amersham). Then, the membrane was placed in a cassette (Radiographic Chassis 30 × 40 cm^2^, Konex, Sao Paulo, SP, Brazil) under a radiographic film measuring 18 × 24 cm^2^ (Agfa Gevaert, NV, Mortsel, Belgium) for approximately 40 min. The film was later developed manually using the conventional temperature–time method, according to the manufacturer’s guidelines. The solutions used were from Kodak (Kodak Brasileira Com. E Ind. Ltda., São José dos Campos, SP, Brazil) and were maintained at a temperature of 20 °C.

A single trained examiner read the radiographic films, who was calibrated and blinded to the treatments used. The readings were performed 2 times on different days to check the results. The intensity of each signal produced by a given probe in the biofilm sample was compared with the signal produced by the same probe in the 2 lines of controls containing 10^5^ and 10^6^ bacteria. Thus, the number 0 was registered when there was no signal detected; 1 was equivalent to a less intense signal than the 10^5^-cell control; 2 was equivalent to 10^5^ cells; 3 was between 10^5^ and 10^6^ cells; 4 was approximately 10^6^ cells; and 5 was more than 10^6^ cells. These records were used to determine the levels of the different species in the different samples evaluated.

### 2.6. Statistical Analysis

Microbiological data were expressed as counts (levels) of the 33 bacterial species assessed. Significant differences between the two groups were evaluated using the Mann–Whitney test. The statistical significance was set to 5%. Statistical analyses were performed with SPSS 11.0 (IBM Corporation, Armonk, NY, USA).

## 3. Results

**Figure 1:**
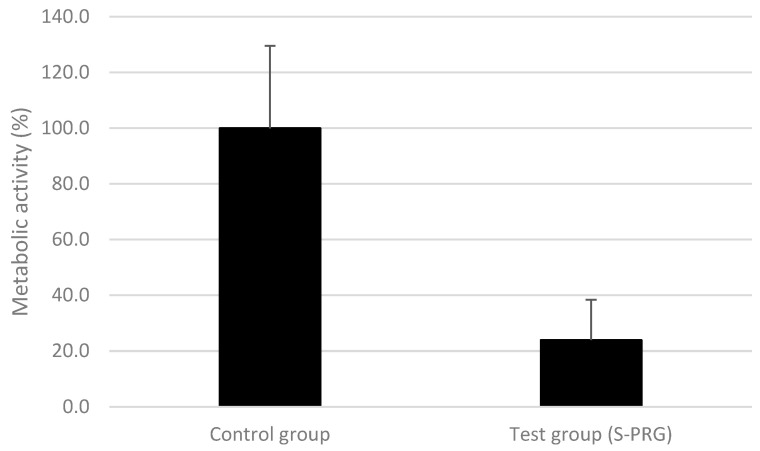
Metabolic activity of the multispecies biofilm formed on the control and test pegs. The control group’s biofilms were considered to have 100% metabolic activity. Statistical significance was detected using the Mann–Whitney test between groups (*p* ≤ 0.05).

Figure 1 shows the results of the metabolic activity of the biofilms formed on the S-PRG pegs and on the control pegs. Metabolic activity is an indirect measure of the viability of the bacterial cells of the biofilms, in which a color change indicates a greater biofilm formation. The test group decreased the metabolic activity by more than 75% compared with the control group (*p* ≤ 0.05), indicating a much lower biofilm formation on the pegs covered with S-PRG than on the uncovered pegs.

**Figure 2:**
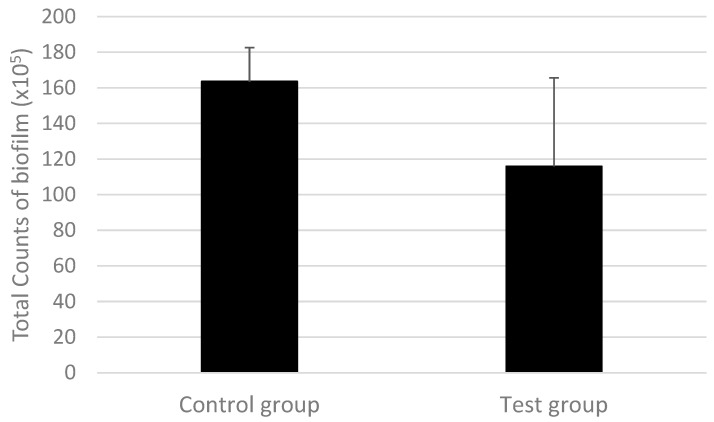
Total counts of the multispecies biofilm formed on control and test groups. Statistical significance was detected using the Mann–Whitney test between groups (*p* ≤ 0.05).

Figure 2 shows the total counts of all microorganisms included in the biofilm experiment. The data (total counts) include the total checkerboard values of all bacterial species included in the model; however, it did not specify the amounts of any species. This indicates the effect of the agent on the entire biofilm. The test group (S-PRG) presented smaller counts (a reduction of approximately 30%) than the values of the control group (*p* ≤ 0.05).

Table 2 shows the proportions of the complexes in each group. Socransky et al. [33] organized some species present in the subgingival milieu into complexes according to their role in developing periodontal disease. The purple, yellow, and green complexes, as well as the Actinomyces group, are associated with health conditions. The presence and abundance of the orange complex are related to the transition from a healthy situation to disease conditions, while the red complex is strongly associated with periodontitis. In this sense, the biofilms formed in the test group presented a statistically significant smaller proportion of the diseased-associated red complex than the biofilms formed in the control group.

**Figure 3:**
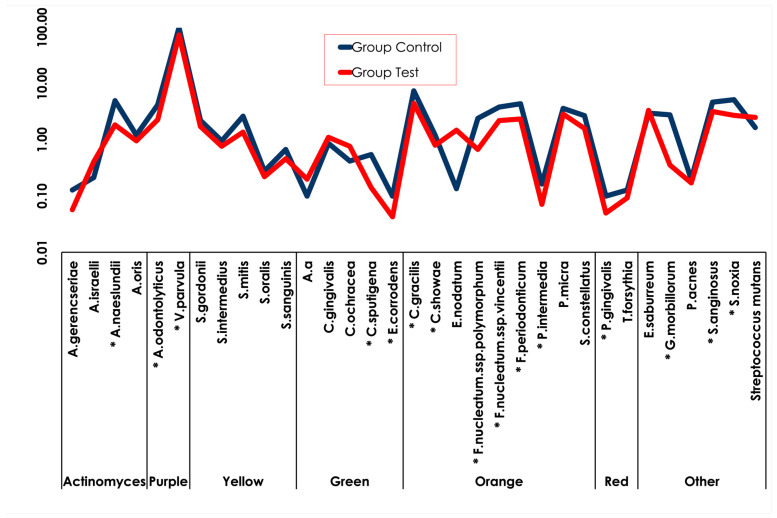
Total counts of the multispecies biofilm formed in the control and test groups. Statistical significance was detected using the Mann–Whitney test between groups (* *p* ≤ 0.05).

Figure 3 shows the average counts of each bacterial strain included in the model (mean counts (×10^5^) of the species evaluated) in the two groups of biofilms. The biofilm formed in the experimental group presented smaller amounts of 15 species compared with the biofilms formed in the control group. The bacterial species reduced in the experimental group included members of the Actinomyces group, as well as the purple, green, orange, and red complexes as follows: *Actinomyces naeslundii*, *Actinomyces odontolyticus*, *Veillonella parvula*, *Capnocytophaga ochracea*, *Capnocytophaga sputigena*, *Eikenella corrodens*, *Campylobacter gracilis*, *Fusobacterium nucleatum polymorphum*, *Fusobacterium nucleatum vincentii*, *Fusobacterium periodonticum*, *Prevotella intermedia*, *Porphyromonas gingivalis*, *Gemella morbillorum*, *Streptococcus anginosus*, and *Selenomonas noxia.* (*p* ≤ 0.05).

## 4. Discussion

The data of this study suggest that an S-PRG barrier coating is effective in reducing the level of bacteria from the yellow and red complexes in a multispecies biofilm in vitro. Probably, the ions released from the S-PRG filler, which provide bioactivity, significantly reduced the counts of several members of the orange complex (*C. gracilis*, *C showae*, *F. nucleatum polymorphum*, *F. nucleatum vincentii*, *F. periodonticum*, and *P. intermedia*) and the counts of *P. gingivalis*, a member of the red complex.

Among the ions released by the S-PRG filler (fluoride, strontium, sodium, boron, aluminum, and silica), fluoride is best known for its ability to reduce demineralization and improve remineralization. An in vitro thermal and acid challenge of the S-PRG barrier coating showed effective prevention against demineralization in the subsuperficial area and higher nanohardness values around the coated area compared with the control group [34]. Microbiological data indicate that fluoride can reduce the production of acid by *S. mutans* through a various number of mechanisms, including inhibition of the glycolytic enzyme enolase and the proton-extruding ATPase, and could also be related to interference with other intracellular or plaque-associated enzymes in *S. mutans*, such as acid phosphatase, pyrophosphatase, peroxidase, and catalase [35].

The release of strontium might result in the formation of strontium–apatite complexes and enhance remineralization of the enamel in conjunction with fluoride [36]. Moreover, fluoride binds to strontium to form an acid-resistant layer and converts hydroxyapatite into a fluoride–apatite complex, which inhibits demineralization [37]. Silicon and aluminum are typical elements of filler glass and might help in the remineralization process [34].

Boron inhibits several biological processes in different ways and is known to provide antibacterial activity against *S. mutans*, *S. aureus*, *E. coli*, *Candida albicans* and periodontopathogenic bacteria, as well as inhibiting quorum sensing in both bacteria and fungi, which is a key factor in the formation of biofilms [20,27]. In rats, systemic administration of boric acid may reduce the loss of alveolar bone due to its interference with the RANKL/OPG balance in periodontal disease [38]. Hakki et al. (2010) suggested that metal ions released from the S-PRG filler might have the ability to wield anti-inflammatory effects and, among the metal ions, boric acid may play an important role in preventing ligature-induced periodontal disease [39,40]. Sodium ions work as a buffer and potentialize the other ions, improving the antibacterial effects.

The S-PRG filler acts in a quasi-intelligent way, as the ions are released by acidity-dependent conditions. Thus, its protective effect is proportional to the threat encountered. Moreover, the main antibacterial effect observed in this study may not be associated with the release of a specific ion but the release of the multiple ion species and their synergy [34].

It has also been suggested that materials with S-PRG filler are able to modulate the effect of acidic solutions, bringing the pH values closer to neutral by releasing ions and preventing the growth of bacteria [34]. Several studies have demonstrated that resin composites containing S-PRG fillers can effectively reduce bacterial adhesion and prevent the formation of biofilms on their surfaces [18,23,25,41].

Several in vitro studies have shown the antibacterial effects of S-PRG filler against *S. mutans*, the major pathogen of dental caries [28]. S-PRG fillers decreased the expression of *S. mutans* genes involved in the metabolism of sugar and also had an inhibitory effect on *S. oralis* and *S. gordonii*. Since it can inhibit the metabolism of sugar by oral *Streptococci*, S-PRG results in the mitigation of cariogenicity, especially before the active growth phase [28]. No statistically significant differences were observed in *S. mutans* or *S. gordonii* in the present study. However, none of the previous research used a controlled multispecies biofilm, as was used in the present study. Moreover, this complex biofilm model focused on a periodontopathogenic biofilm, where the interaction of *S. mutans* and *S. gordonii* with other anaerobic bacteria might differ from that found in less complex microbiological studies performed on planktonic bacteria or single-species biofilms [37]. In addition, the findings suggest that an S-PRG filler containing bioactive materials can be beneficial not only for the prevention of dental caries, but can also interfere with the homeostasis of buccal microflora. The suppression of bacterial activity is considered a key modality in controlling dental caries and periodontal diseases.

A limitation of the present study is the absence of a positive control. However, the majority of the other materials release only fluoride, and the literature has already demonstrated its weak antibacterial action. The anticaries action of fluoride is due to a physicochemical mechanism that acts by inhibiting demineralization but is not due to antibacterial activity. Since we focused on periodontal disease, the action of demineralization was not relevant here.

The checkerboard DNA–DNA hybridization technique was chosen for its ability to evaluate 33 species in our complex biofilm [2,5,42]. It showed a reduction in the amount of the periodontopathogenic species, *P. gingivalis*, and some *Fusobacterium* species. Yoneda et al. (2012) showed that an eluate of S-PRG inhibited the protease and gelatinase activities of *P. gingivalis* and suppressed coaggregation between *P. gingivalis* and *Fusobacterium* [25]. Another study showed that an eluate of S-PRG inhibited the formation of biofilm and disrupted mature biofilms [31]. *P. gingivalis* is a Gram-negative periodontopathogenic bacterium that is unable to metabolize sugar and uses protease activity to degrade proteins as energy sources. S-PRG fillers can inhibit the protease activity of *P. gingivalis*. Kono et al. speculated that *P. gingivalis* may suffer from a process of oxidative stress generated by the eluate of S-PRG, which causes considerable damage and acts to suppress bacterial growth and their virulence factors, especially bacterial adhesion factors and the production of protease [31].

*Fusobacterium* counts were also reduced in this study. This is a Gram-negative species related to the accumulation of mature dental plaque, which is in agreement with Kono et al.’s data [31]. An eluate of S-PRG was found to have a suppressive effect on the BAPNA-hydrolyzing and gelatinase activities of *P. gingivalis*. The coaggregation of periodontopathic bacteria is associated with bacterial attachment in the gingival crevice [43].

The literature shows that an eluate of S-PRG suppresses streptococcal adherence, which is a key factor in the development of caries as it inhibits the protease and coaggregation activities of *P. gingivalis* as a key factor of periodontal disease. These findings may lead to a search for new strategies to prevent caries and periodontal diseases [25].

Furthermore, S-PRG filler was reported to exert oxidative stress on Candida species, inhibiting their adhesive properties and production of proteinase, and, consequently, their pathogenicity. Candida species are involved in opportunistic infections and systemic diseases in the elderly population [28,44].

Other variables observed in this study included the cell metabolism of bacteria in the biofilms. The metabolism of bacteria within bacterial biofilms is commonly used as a quantitative parameter [45,46]. The results of this study showed a statistically significant reduction in the total counts of bacteria in the biofilm in the S-PRG group, which had approximately 30% fewer bacteria than the control group (Figure 2) and also a statistically significant reduction in metabolic activity of 70% in the test group (Figure 1). In contrast, Komalsingsakul et al. observed no difference in live/dead mutant biofilms either within or between the S-PRG composite, glass ionomer, or composite resin groups, even though the average of absolute surface roughness and the release of fluoride ions were significantly different [47]. This is opposed to a study by Nedeljkovic et al., which demonstrated the strong inhibitory effect of glass ionomer materials and giomers on the growth of *S. mutans* [13]. In addition, the duration of bacterial growth must be considered. Suzuki et al. observed that for salivary bacteria, a short incubation time was unable to prevent bacterial growth; however, following a longer incubation, all viable bacterial counts in the saliva were lower than in the untreated controls [20]. Kim et al. observed the antimicrobial and antibiofilm effects of an S-PRG filler in a multibacterial model obtained from human saliva [37]. Treatment with an eluate of S-PRG was applied for a short time (2 × 5 min) and no effects were observed. However, a longer time (as used in the present study) with a known biofilm species did show the antibacterial and antibiofilm effects of the S-PRG filler. Furthermore, the evaluation of differences in the frequency and quantity of the pathogens is critical in identifying the relationship between periodontopathogenic bacteria and periodontal disease.

The trigger for the initiation of periodontal diseases is the presence of complex microbial biofilms. A complex consisting of *P. gingivalis* and *T. forsythia*, which is called the red complex, plays important roles in the pathogenesis of periodontal disease and is highly related to clinical parameters, such as the depth of the periodontal pocket and bleeding on probing [48]. A second group of bacterial species known as the orange complex, which includes *C. gracilis*, *C showae*, *F. nucleatum polymorphum*, *F. nucleatum vincentii*, *F. periodonticum*, and *P. intermedia*, is also associated with the clinical parameters of disease. Microorganisms from both complexes generally act together and evidence has shown that colonization by species from the red complex is preceded by colonization by, and proliferation of, the orange complex [33]. Many species from the yellow complex, as well as *P. gingivalis* from the red complex, were significantly reduced by the S-PRG barrier coating. Thus, it is possible that impairing the development of the yellow and red complexes could have some clinical benefits against periodontitis. In addition, as periodontitis is an inflammatory disease, Iwamatsu-Kobayashi et al. showed that an eluate of S-PRG prevented the destruction of tissue in experimental periodontal disease through anti-inflammatory effects [40]. Yamaguche et al. showed that the eluate of S-PRG was able to promote the migration of HGF-1 fibroblast cells, which may help in tissue repair [49]. Moreover, the buffering ability of supragingival S-PRG fillers resulted in a more favorable proportion of bacterial species related to health. The bioactive properties of S-PRG fillers in association play an important role in maintaining oral health and must be considered for treating high-risk patients.

In summary, this is the first study showing the effect of an S-PRG barrier coating on a complex subgingival multispecies biofilm model, and we suggest that the S-PRG barrier coating has an antibacterial effect against several periodontal pathogens, highlighting its effects on *P. gingivalis*. Clinical data must be produced to ensure the effects of the material tested here. These findings may encourage research into novel strategies with bioactive materials for preventing periodontitis.

## 5. Conclusions

Within the limitations of this study, our results suggest that the S-PRG barrier coating has an antibacterial effect against 15 out of the 31 periodontal pathogens evaluated, highlighting its effects on *P. gingivalis*. These findings suggested that the bioactive material could be helpful in treating patients with a history of periodontitis and non-carious gingival recession after surgical treatment.

## Figures and Tables

**Table 1:** Composition of the multispecies biofilm, with the list of bacteria cultured and categorized into the microbial complexes described by Socransky et al. [33].

Strains in the Multispecies Biofilm
**Actinomyces complex**
*Actinomyces gerencseriae* (ATCC 23840)
*Actinomyces israelii* (ATCC 12102)
*Actinomyces oris* (ATCC 43146)
*Actinomyces naeslundii* (ATCC 12104)
**Purple complex**
*Actinomyces odontolyticus* (ATCC 17929) *Veillonella parvula* (ATCC 10790)
**Yellow complex**
*Streptococcus intermedius* (ATCC 27335)
*Streptococcus mitis* (ATCC 49456)
*Streptococcus gordonii* (ATCC 10558)
*Streptococcus oralis* (ATCC 35037)
*Streptococcus sanguinis* (ATCC 10556)
**Green complex** *Aggregatibacter actinomycetemcomitans* (ATCC 29523)
*Capnocytophaga ochracea* (ATCC 33596)
*Capnocytophaga gingivalis* (ATCC 33624)
*Capnocytophaga sputigena* (ATCC 33612)
*Eikenella corrodens* (ATCC 23834)
**Orange complex** *Campylobacter showae* (ATCC 51146) *Campylobacter gracilis* (ATCC 33236) *Eubacterium nodatum* (ATCC 33099)
*Fusobacterium nucleatum vincentii* (ATCC 49256)
*Parvimonas micra* (ATCC 33270)
*Fusobacterium nucleatum polymorphum* (ATCC 10953)
*Fusobacterium periodonticum* (ATCC 33693)
*Prevotella intermedia* (ATCC 25611) *Streptococcus constellatus* (ATCC 27823)
**Red complex**
*Porphyromonas gingivalis* (ATCC 33277)
*Tannerella forsythia* (ATCC 43037)
**Other**
*Gemella morbillorum* (ATCC 27824)
*Propionibacterium acnes* (ATCC 11827)
*Selenomonas noxia* (ATCC 43541)
*Streptococcus anginosus* (ATCC 33397)
*Streptococcus mutans* (ATCC 25175)

**Table 2:** Average proportions of bacterial complexes [33]. The blue group comprises four *Actinomyces* species.

Complex	Control Group Mean ± SD	Test Group Mean ± SD	*p*-Value Mann–Whitney
Actinomyces	4.02 ± 0.46	3.54 ± 2.35	0.511
Purple	64.41 ± 6.52	60.24 ± 22.62	0.517
Yellow	3.94 ± 2.77	3.9 ± 3.68	0.874
Green	1.24 ± 0.46	2.86 ± 3.86	0.227
Orange	15.71 ± 3.50	18.67 ± 12.79	0.427
Red	0.14 ± 0.15	0.1 ± 0.18	0.039
Other	10.54 ± 2.81	10.69 ± 6.14	0.936

## Data Availability

Not applicable.
